# Novel and nano-rare genetic causes of paediatric-onset motor neuronopathies

**DOI:** 10.1093/braincomms/fcae003

**Published:** 2024-01-05

**Authors:** Arman Cakar, Reza Maroofian, Yesim Parman, Mary M Reilly, Henry Houlden

**Affiliations:** Neuromuscular Unit, Istanbul University, Istanbul Faculty of Medicine, Istanbul 34093, Turkey; Department of Neuromuscular Disorders, UCL Queen Square Institute of Neurology, London WC1N 3BG, UK; Department of Neuromuscular Disorders, UCL Queen Square Institute of Neurology, London WC1N 3BG, UK; Neuromuscular Unit, Istanbul University, Istanbul Faculty of Medicine, Istanbul 34093, Turkey; Department of Neuromuscular Disorders, UCL Queen Square Institute of Neurology, London WC1N 3BG, UK; Department of Neuromuscular Disorders, UCL Queen Square Institute of Neurology, London WC1N 3BG, UK

We read the article by Zambon *et al*.^1^ regarding paediatric-onset motor neuropathies with great interest. The authors described clinical details and novel genetic findings with associated molecular mechanisms in this rapidly evolving field. Around 30 causative genes have been identified to give rise to distal hereditary motor neuropathies (dHMN). The inheritance pattern can be autosomal dominant, recessive or X-linked, increasing these disorders’ genetic complexity. Only ∼30% dHMN patients receive a genetic diagnosis despite the advances in molecular genetics. The phenotypical presentation of pure dHMN classified by Harding *et al*.^2^ is relatively uniform. On the other hand, significant overlap between Charcot–Marie Tooth type 2 (CMT2), complicated hereditary spastic paraplegia (HSP), juvenile forms of amyotrophic lateral sclerosis (jALS) and distal myopathies can cause terminological confusion. In this regard, Zambon and colleagues used ‘motor neuronopathies’ term, which encompasses dHMN, CMT2 overlap group with minor sensory involvement as well as spinal muscular atrophy with lower extremities predominance, arthrogryposis multiplex congenita, jALS and motor neuropathies with central nervous system involvement. These broad spectra of clinical phenotypes cover 55 causative genes, with autosomal dominant (17/55), recessive (31/55), X-linked (4/55) and dual (3/55) transmission.^1^ In this letter, we aimed to broaden the genetic spectrum by analysing the novel or nano-rare genetic causes resulting in various subtypes of motor neuronopathies and provide an update for the recent reviews in this evolving field.^1,3^

## RNA binding proteins

Biallelic *GEMIN5* variants were described to cause a developmental delay with a combination of additional clinical features such as motor neuronopathy, pyramidal signs and cerebellar dysfunction that started the first two years of life in 30 patients (Table 1, Supplementary material). GEMIN5 is a highly conserved multifunctional protein that interacts with several RNA and protein targets, including survival motor neuron (SMN), a vital protein for motor neurons. Loss of SMN protein causes spinal muscular atrophy, a progressive and fatal motor neuron disease. Loss of function (LoF) variants in *GEMIN5* perturbed small nuclear ribonucleoproteins (snRNPs) assembly and misregulated RNA targets.

**Table 1:** Clinical features of paediatric-onset motor neuronopathies

	GEMIN5^a^	EXOSC9^a^	RBM7^a^	TRIP4^a^	ASCC1^a^	ADPRHL^a^	NRCAM^a^	MME^a^	UBE3C^a^	SLC5A6^a^	SLC25A2^a^	BANF1^a^	NAGLU^a^
Number of cases	>10 from different families	10 from different families	1	6 from 4 families^b^	5 from 4 families^b^	3^c,d^	2^c,d^	1^c^	9 from single family	5 from 3 families	1	1	1
Onset of neuronopathy	Birth–1st decade	Birth–3 years	1 month	Birth	Birth	2nd decade^c^	2nd decade	16 years^c^	3–40 years	1st–2nd decade	3 years	3 years	2 years and 6 months
NDD	±	+	+	+	+	−	±	−	−	−	−	−	−
Hypotonia	±	±	+	+	+	−	−	−	−	−	−	−	−
Weakness	Generalized	Generalized	Generalized	Generalized	Generalized	Distal predominant	Distal predominant	Distal predominant	Distal predominant	Distal or generalized	Distal	Generalized	Generalized
Pyramidal signs	±	−	−	−	−	±	−	−	±	±	±	−	−
Skeletal deformities	CMA ± claw hands	CMA ± contractures ± valgus deformity	-	CMA ± multiple fractures	CMA ± multiple fractures ± talipes equinovarus ± contractures	Pes cavus ± scoliosis	Pes cavus ± hammer toes ± scoliosis ± claw hands	Pes cavus ± contractures	Pes cavus ± hammer toes	-	Claw hands, scoliosis	Scoliosis	-
Respiratory involvement	±	−	+	+	+	±	−	−	−	−	+	+	−
Epilepsy/ID	+/+	+/+	−	−	−	+/+	−	−	−	−	−	−	−
Disease course	Static or progressive	Progressive	Progressive	Progressive	Progressive	Progressive	NA	NA	NA	Progressive	Progressive	Progressive	Slowly progressive
Allelic disorders	PCH	PCH1D	-	CM	CM	CONSDIAS	NDD	CMT2T, SCA43	Angelman-like syndrome	SMVTD	-	NGPS	CMT2V, MPS3B
Variants	Missense, LoF	Missense, LoF	Missense	Missense, LoF	LoF, deletion	Missense	Missense, LoF	Missense, in-frame deletion	CSV	Missense, LoF	Missense	Missense	Missense

CM, congenital myopathy; CMA, congenital multiplex arthrogryposis; CMT, Charcot–Marie–Tooth disease; CONSDIAS, stress-induced childhood-onset neurodegeneration with variable ataxia and seizures syndrome; CSV, complex structural variant; ID, intellectual disability; LoF, loss of function; MPS, mucopolysaccharidosis 3B; NA, not applicable; NDD, neurodevelopmental disorder; NGPS, Néstor–Guillermo progeria syndrome; SMVTD, sodium-dependent multivitamin transporter deficiency; SCA, spinocerebellar ataxia; PCH, pontocerebellar hypoplasia; PCH1D, pontocerebellar hypoplasia 1D.

^a^References for the table can be found in the Supplementary material.

^b^Cases described as congenital myopathy are excluded.

^c^Later-onset cases are excluded.

^d^Cases with neuropathy as a part of complex disease spectrum are excluded.

## Exosome complex subunits

Homozygous or compound heterozygous variants in the *EXOCS9*, encoding a subunit of exosome complex, were described to cause pontocerebellar hypoplasia (PCH) in four patients from different families (Table 1, Supplementary Table 1). Following the initial publication, six more patients from different groups with different biallelic variants in the *EXOCS9* were reported (Table 1, Supplementary Table 1). Exosome complex has various functions regarding gene expression by modulating RNA decay. Other genes encoding subunits of the exosome complex, *EXOSC3* and *EXOSC8*, were also described to cause PCH, spinal muscular atrophy (SMA) and central nervous system demyelination.^1^ Moreover, a homozygous missense variant in the *RBM7*, a co-factor of the exosome complex, was shown in an infant with a progressive disease course resulting in death (Table 1, Supplementary Table 1).

## Transcriptional regulators

Pathogenic variants in *TRIP4* and *ASCC1*, subunits of activating signal co-integrator 1 (ASC-1), were shown to cause prenatal-onset spinal muscular atrophy with congenital bone fractures (SMABF) type 1 and 2, respectively (MIM#616866, MIM#616867). The typical presentation is severe hypotonia and respiratory distress (Table 1, Supplementary Table 1). Intriguingly, muscle biopsy of patients showed a primary myopathic process and detailed electrodiagnostic and radiological studies are currently lacking to conclude the neuronal role in muscle weakness. Furthermore, later-onset myopathy with mild clinical course was also described in patients with *TRIP4* and *ASCC1* variants, suggesting a broad phenotypical presentation regarding age at onset and neuromuscular phenotype.^4^ Interestingly, patients with *VWA1* variants may also present with dHMN with myopathic features.^5^

## ADP-ribosylation enzymes

Recessive pathogenic variants in *ADPRHL2* give rise to CONDSIAS (stress-induced childhood-onset neurodegeneration with variable ataxia and seizures syndrome). ADP-ribosylation is a vital process in post-translational modification and requires precise control. ARH3, an (ADP-ribosyl) hydrolase encoded by *ADPRHL2*, is one of the critical enzymes in the process. Although the phenotypical features in CONSDIAS cover multiple domains of the nervous system and other organs, neuropathy (motor > sensory) predominant cases with various age of disease onset were also described^6^ (Table 1, Supplementary Table 1).

## Transmembrane proteins

Biallelic variants in *NRCAM* encoding a highly expressed cell adhesion protein in the nervous system were described to cause a neurodevelopmental disorder (NDD) with developmental delay, intellectual disability, hypotonia, spasticity and peripheral neuropathy. Interestingly, two patients in this report had isolated motor neuropathy phenotype, with absent or minimal additional symptoms (Table 1, Supplementary Table 1). Subsequently, another patient with motor-predominant polyneuropathy was reported (Table 1, Supplementary Table 1). To date, variants in other genes encoding cell adhesion protein such as *L1CAM* and *NFASC* were described to cause several syndromes such as HSP (MIM#303350, MIM#307000) and NDD with peripheral motor dysfunction (MIM#609145), respectively.

*MME* encodes membrane metalloendopeptidase, a neutral transmembrane endopeptidase also named neprilysin. Variants in *MME* are well-described causes of CMT2, and dHMN. Previously described cases predominantly had a disease onset after the fourth decade. On the other hand, Hong *et al*.^7^ described a juvenile-onset case, compound heterozygous for c.1416+2T>C (p.Val440_Lys472*) and c.2027C>T (p.Prp676Leu) variants, suggesting the age of onset in MME-related neuropathy may rarely present earlier than current knowledge (Table 1, Supplementary Table 1).

## Ubiquitin-proteosome system

A novel structural variant caused by 1.35 Mb complex insertion that contains four different protein-coding genes (*HB9*, *NOM1*, *RNF32*, *LMBR1*), their regulatory elements, with upstream regulatory elements, and the first 10 exons of the *UBE3C* ligase gene was described as a novel cause of dHMN in an Australian family^8^ (Table 1, Supplementary Table 1). *UBE3C* intergenic fusion (UBE3C-IF) resulting from the structural variant was resistant to nonsense-mediated decay and caused a reduction of wild-type UBE3C protein in the spinal motor neuron model reprogrammed from patient-derived induced pluripotent stem cell. Furthermore, transgenic *Caenorhabditis elegans* expressing the UBE3C-IF transcript in GABAergic motor neurons exhibited neuronal synaptic transmission deficits and susceptibility to heat stress. Although the authors did comprehensive functional analyses, further families are needed to confirm the role of *UBE3C* in dHMN phenotype since the structural variant may have various other effects, such as gene dosage changes or ectopic expression of other flanking genes, as suggested. On the other hand, *UBE1*, encoding another enzyme in the ubiquitin pathway that initiates the activation and conjugation of ubiquitin causes of X-linked infantile SMA, further supporting the pathogenicity of *UBE3C*. Intriguingly, biallelic LoF variants were recently shown to cause more severe, Angelman-like syndromic NDD with seizures, movement disorders and neurobehavioral abnormalities, suggesting that *UBE3C*-related disorders may have a broad phenotypical spectrum.^9^

## Ion channel proteins and transporters

Variants in *SLC5A6* were associated with multisystemic disorders, including failure to thrive, developmental delay, polyneuropathy, seizures, cerebral palsy, gastrointestinal problems, immunodeficiency and osteopenia. In 2022, five patients from three families were identified to carry biallelic variants in the *SLC5A6* gene with a phenotype compatible with motor neuropathy (Table 1, Supplementary Table 1). Sodium multivitamin transporter is encoded by *SLC5A6* and necessary for sodium-dependent uptake of biotin and pantothenic acid, α-lipoic acid and iodide. The authors suggested that reduced catalytic activity of the enzyme resulting in decreased uptake of biotin and alpha-lipoic acid may contribute to neuropathy phenotype.

Furthermore, a homozygous variant in the *SLC25A21* gene was described to cause a spinal muscular atrophy-like phenotype accompanied by mitochondrial myopathy in a 19-year-old female (Table 1, Supplementary Table 1). SLC25A21 is a member of the mitochondrial carrier family working as a transporter across the mitochondrial inner membrane. Defects in mitochondrial carrier family members are associated with various disorders, including rhabdomyolysis, lipid storage myopathy, neuropathy, epileptic encephalopathy, optic atrophy and cardiomyopathy.

## Nuclear envelope components

A rare homozygous p.Ala12Thr variant in the *BANF1* gene, encoding barrier-to-autointegration factor (BAF), causes Néstor–Guillermo progeria syndrome, a premature aging syndrome. BAF is a highly conserved metazoan chromatin protein essential for nuclear organization. In 2023, a *de novo* heterozygous *BANF1* variant, p.Gly16Arg, was identified in an 8-year-old girl with progressive neuromuscular weakness (Table 1, Supplementary Table 1). Further functional analysis for the effect Gly16Arg showed a structural change, increasing the DNA binding affinity of BAF, which changed the transcriptional and epigenetic features of the cell. Pathogenic variants in *VRK1*, a gene encoding serine/threonine kinase that phosphorylates BAF, causes neurological disorders ranging from pontocerebellar hypoplasia clinical similarities of BANF1-related disease to dHMN with (MIM#607596, #620542). Furthermore, *LMNA* encoding a nuclear envelope partner of BAF, lamin A/C gives rise to ARCMT2B1 (MIM#605588), supporting the role of *BANF1* in motor neuronopathies.

## Lysosomal enzymes

*NAGLU* encodes an enzyme that degrades heparan sulphate, a ubiquitously expressed glycosaminoglycan. Biallelic variants in this gene give rise to mucopolysaccharidosis type IIIB, whereas heterozygous variants result in late-onset sensorimotor neuropathy (CMT2V). Regarding motor neuropathy, a single case was published with symptoms starting at 30 months with a novel heterozygous c.1435G>A (p.Ala479Thr) variant in the *NAGLU* gene (Table 1, Supplementary Table 1). The patient showed partially reduced urinary alpha-*N*-acetylglucosaminidase activity compared to severe disease form with a nearly total loss of enzyme activity. Lysosomal storage disorders, such as Fabry disease (MIM#301500), Metachromatic Leukodystrophy (MIM#250100) and GM2 Gangliosidosis (MIM#272800, #268800), are also well-described causes of peripheral neuropathies making *NAGLU* a more interesting gene to focus on as a cause of dHMN phenotype.

**Figure 1:**
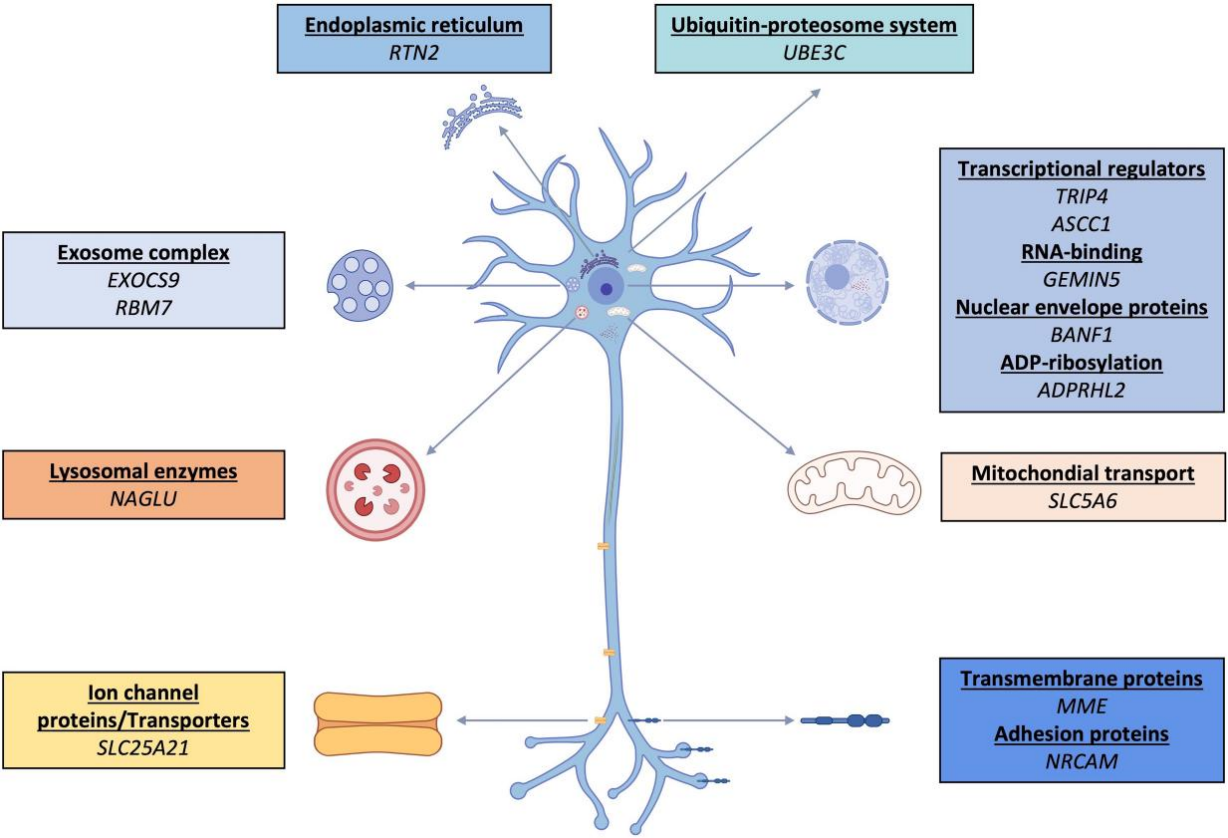
**Schematic representation of a motor neuron.** Note the diversity of the cellular mechanisms involved in motor neuronopathy pathology. Created with BioRender.com.

The review of Zambon *et al*.^1^ with our report demonstrates that the genetic spectrum of motor neuronopathies covers many different genes in different pathways with significant overlap between various neurological disorders (Fig. 1). Apart from the above, the motor neuronopathy term also encompasses *SOD1*, *ERLIN1*, *TARDB* and *GNE* genes, which were rarely described in jALS patients.^10^ Moreover, phenotypic expansion in known genes is frequently reported with the widespread use of next-generation sequencing technologies. For instance, *SPG15* and *SPTAN1* variants, which were initially described to cause distinct neurodegenerative syndromes, were also observed in cases with dHMN.^11,12^ One can easily suggest that the landscape will continue to evolve with recently identified genes, such as *COQ7* and *RTN2* (unpublished data), to cause various types of motor neuronopathies on the horizon.^1^ Despite the rapid advances in molecular genetics, the discovery of new genes, ultra and nano-rare variants, and singleton families complicate genetic diagnosis. Indeed, most genes mentioned in our letter were shown only in single families, and other affected families are needed to confirm the causal relationship. On the other hand, increasing data regarding the disease mechanisms caused by novel genetic causes pave the path for understanding nerve biology and associated phenotypes, eventually supporting the identification of disease-modifying therapeutic interventions.

## Supplementary Material

fcae003_Supplementary_Data

## Data Availability

Data sharing is not applicable to this article as no new data were created or analysed.
