# Supplementary material for: Novel and nano-rare genetic causes of paediatric-onset motor neuronopathies
Source: Brain Commun. 2024 Jan 5;6(1):fcae003. doi: 10.1093/braincomms/fcae003 (PMC10808011; doi:10.1093/braincomms/fcae003)
Supplement: fcae003_Supplementary_Data [file fcae003_supplementary_data.pdf]

**Supplementary Table 1:** Detailed phenotypical and genetic features of paediatric-onset motor neuronopathies.

| | ***GEMIN5*** *Kour et al, 2021*[1] | ***EXOSC9*** *Burns et al, 2018*[2] *Bizzari et al, 2019*[3] *Sakamoto et al, 2021*[4] *Dabaj et al, 2022*[5] | ***RBM7*** *Giunta et al, 2016*[6] | ***TRIP4*** *Knierim et al, 2016*[7] *Töpf et al, 2021*[8] | ***ASCC1*** *Knierim et al, 2016*[7] *Giuffrida et al, 2020*[9] *Lu et al, 2020*[10] *Rosano et al, 2021*[11] | ***ADPRHL2*** *Beijer et al 2021*[12] | ***NRCAM*** *Kurolap et al, 2022*[13] *Elahi et al, 2023*[14] | ***MME*** *Hong et al, 2019*[15] | ***UBE3C*** *Gopinath et al, 2007*[16] | ***SLC5A6*** *Holling et al, 2022*[17] | ***SLC25A21*** *Boczonadi et al, 2018*[18] | ***BANF1*** *Marcelot et al, 2023*[19] | ***NAGLU*** *Lopergolo et al, 2023*[20] |
|---|---|---|---|---|---|---|---|---|---|---|---|---|---|
| **Number of cases** | >10 from different families | 10 from different families | 1 | 6 from 4 families[c] | 5 from 4 families[c] | 3[a, b] | 2[b] | 1[a] | 9 from single family | 5 from 3 families | 1 | 1 | 1 |
| **Age at onset of neuropathy symptoms** | Birth – 1st decade | Birth – 3 years | 1 month | Birth | Birth | 2nd decade[a] | 2nd decade | 16 years[a] | 3 – 40 years | 1st – 2nd decade | 3 years | 3 years | 2 years and 6 months |
| **Initial symptoms** | Developmental delay ± hypotonia | Developmental delay ± hypotonia | Hypotonia, poor sucking, failure to thrive | Arthrogryposis multiplex congenita, congenital fractures and neonatal respiratory distress | Arthrogryposis multiplex congenita, congenital fractures and neonatal respiratory distress | Walking instability, ± fatigue ± atrophy of intrinsic hand muscles distal lower limbs | Distal lower limb weakness | Lower limb weakness | Walking and running difficulties due to lower limb weakness | Fine motor difficulties ± slow walking | Deteriorated walking, frequent falls | Abnormal and clumsy gait | Weakness in upper and lower limbs |
| **Muscle strength** | Generalized weakness | Proximal and distal weakness (1 year) | Generalized weakness | Generalized weakness, hypotonia | Generalized weakness, hypotonia | Distal predominant generalized weakness | Distal or distal-predominant weakness | Distal and lower extremity predominant weakness (24 years) | Distal (predominantly in upper extremities) or generalized weakness | Weakness and atrophy in hands, foot drop, proximal muscles are normal (19 years) | Distal weakness | Diffuse muscle weakness that affected legs more than arms, a complete foot drop (8 years) | Widespread weakness, Atrophy of distal parts of extremities (60 years) |
| **Pyramidal signs** | Hyperreflexia | - | - | - | - | ± (spasticity) | - | - | Rarely (Brisk reflexes, Babinski sign) | Brisk reflexes, Achilles clonus | Brisk reflexes, Achilles clonus | - | - |
| **Sensory signs** | NA | - | - | NA | NA | Hypoesthesia in tip toes, diminished position, and vibration | - | - | - | - | - | Slightly decreased cold sensation of toes | Paresthesia, thermal and painful hypoesthesia in limbs |

| | ***GEMIN5*** *Kour et al, 2021*[1] | ***EXOSC9*** *Burns et al, 2018*[2] *Bizzari et al, 2019*[3] *Sakamoto et al, 2021*[4] *Dabaj et al, 2022*[5] | ***RBM7*** *Giunta et al, 2016*[6] | ***TRIP4*** *Knierim et al, 2016*[7] *Töpf et al, 2021*[8] | ***ASCC1*** *Knierim et al, 2016*[7] *Giuffrida et al, 2020*[9] *Lu et al, 2020*[10] *Rosano et al, 2021*[11] | ***ADPRHL2*** *Beijer et al 2021*[12] | ***NRCAM*** *Kurolap et al, 2022*[13] *Elahi et al, 2023*[14] | ***MME*** *Hong et al, 2019*[15] | ***UBE3C*** *Gopinath et al, 2007*[16] | ***SLC5A6*** *Holling et al, 2022*[17] | ***SLC25A21*** *Boczonadi et al, 2018*[18] | ***BANF1*** *Marcelot et al, 2023*[19] | ***NAGLU*** *Lopergolo et al, 2023*[20] |
|---|---|---|---|---|---|---|---|---|---|---|---|---|---|
| | | | | | | sense in lower legs and hands | | | | | | | |
| **Additional neurological features** | Tremor ± ID ± head nodding ± ataxia ± epilepsy ± micro-cephaly ± respiratory distress ± oculomotor apraxia | Seizures ± psycho-motor regression ± tongue fasciculation ± respiratory insufficiency ± nystagmus | Respiratory difficulties | May result in stillbirth | May result in stillbirth | ID ± seizures ± nystagmus ± postural tremor ± restrictive pulmonary function | - | Postural tremor | - | Tremor ± cerebellar signs | Mild facial weakness, tongue fascic-ulations, decreased FVC | Swallowing and respiratory difficulties | - |
| **Skeletal deformities** | CMA ± claw hands | CMA ± joint contract-ures ± valgus deformities | - | CMA ± multiple fractures | CMA ± multiple fractures ± talipes equinovarus ± joint contract-ures | Pes cavus ± scoliosis | Pes cavus ± hammer toes ± scoliosis ± hyper-lordosis ± claw hands | Pes cavus ± joint contract-ures | Pes cavus ± hammer toes | - | Claw hands, scoliosis | Scoliosis | - |
| **Systemic features** | High-arched narrow palate | Facial dysmorfism ± short stature ± bone fractures ± micro-cephalia ± failure to thrive ± strabismus ± esotropia | Cachexia | Pleural effusion and chylothorax, biliary calculi and soft tissue emphysema | Tent-shaped mouth, micro-retro-gnathia, polyhydro-amniosis, cardio-myopathy, reduced prenatal movements | Growth retardation ± left ventricle hypertrophy ± mitral insufficiency ± micro-gnathia ± hyper-hidrosis ± absent trunk hair | Cataracts | - | - | Dilated cardio-myopathy ± inguinal hernia | Failure to thrive | Poor weight gain | - |

| | ***GEMIN5*** *Kour et al, 2021*[1] | ***EXOSC9*** *Burns et al, 2018*[2] *Bizzari et al, 2019*[3] *Sakamoto et al, 2021*[4] *Dabaj et al, 2022*[5] | ***RBM7*** *Giunta et al, 2016*[6] | ***TRIP4*** *Knierim et al, 2016*[7] *Töpf et al, 2021*[8] | ***ASCC1*** *Knierim et al, 2016*[7] *Giuffrida et al, 2020*[9] *Lu et al, 2020*[10] *Rosano et al, 2021*[11] | ***ADPRHL2*** *Beijer et al 2021*[12] | ***NRCAM*** *Kurolap et al, 2022*[13] *Elahi et al, 2023*[14] | ***MME*** *Hong et al, 2019*[15] | ***UBE3C*** *Gopinath et al, 2007*[16] | ***SLC5A6*** *Holling et al, 2022*[17] | ***SLC25A21*** *Boczonadi et al, 2018*[18] | ***BANF1*** *Marcelot et al, 2023*[19] | ***NAGLU*** *Lopergolo et al, 2023*[20] |
|---|---|---|---|---|---|---|---|---|---|---|---|---|---|
| **Disease course** | Static or progressive course | Progressive (3 patients died during the follow-up between 1.5 and 10 years) | Progressive (Died due to respiratory decompens-ation) | Progressive (lethal in first 2 years), later onset myopathy cases are described | Progressive (lethal in first 2 years), later onset myopathy cases are described | Progressive | NA | NA | NA | Progressive | Progressive course, unable to stand at 18 years | Progressive (Wheelchair use at 10 years) | Slowly progressive |
| **EMG / Nerve conduction studies** | Severe neurogenic pattern, motor neuropathy ± SN ± myopathic pattern | Motor neuron-opathy | Low CMAPs with chronic early and late re-innervation | NA | NA | Axonal motor poly-neuropathy ± mild SN | Segmental neurogenic involvement in L1-S2 and C8-D1, and myopathic motor unit potentials ± SN | Length-dependent motor neuropathy | Slowed tibial conduction in the older patients (range 37–50 m/s) with normal CMAPs | Motor neuropathy ± neuron-opathy ±, mixed demyelin-ating and axonal neuropathy | Severe neurogenic changes | Neurogenic process with diffuse fibrillations and reduced motor responses | Bilateral chronic motor denervation on C7-T1 myotomes |
| **Cranial MRI** | Cerebellar or ponto-cerebellar atrophy ± white matter changes | Cerebellar atrophy ± cerebral atrophy ± white matter atrophy | Normal | Hypoplastic cerebellum, necrosis, haem-orrhage of occipital cortex | Abnormal cortical gyration, lateral ventricle dilatation, simplified gyral pattern of the frontal lobes and enlargement of the external CSF spaces | Normal or mild white matter hyper-intensity lesions | Normal or arachnoid cyst of the posterior fossa | NA | Normal | NA | Normal | NA | NA |
| **Nerve biopsy** | NA | NA | NA | Normal myelin-ization but with loss of unmyelinate | NA | NA | NA | NA | Chronic axonal neuropathy | Normal | Axonopathy with mild and patchy loss of | NA | NA |

| | ***GEMIN5*** *Kour et al, 2021*[1] | ***EXOSC9*** *Burns et al, 2018*[2] *Bizzari et al, 2019*[3] *Sakamoto et al, 2021*[4] *Dabaj et al, 2022*[5] | ***RBM7*** *Giunta et al, 2016*[6] | ***TRIP4*** *Knierim et al, 2016*[7] *Töpf et al, 2021*[8] | ***ASCC1*** *Knierim et al, 2016*[7] *Giuffrida et al, 2020*[9] *Lu et al, 2020*[10] *Rosano et al, 2021*[11] | ***ADPRHL2*** *Beijer et al 2021*[12] | ***NRCAM*** *Kurolap et al, 2022*[13] *Elahi et al, 2023*[14] | ***MME*** *Hong et al, 2019*[15] | ***UBE3C*** *Gopinath et al, 2007*[16] | ***SLC5A6*** *Holling et al, 2022*[17] | ***SLC25A21*** *Boczonadi et al, 2018*[18] | ***BANF1*** *Marcelot et al, 2023*[19] | ***NAGLU*** *Lopergolo et al, 2023*[20] |
|---|---|---|---|---|---|---|---|---|---|---|---|---|---|
| | | | | d axons as documented by "empty" pouches | | | | | | | myelinated fibres | | |
| **Other investigations** | Abnormal EEG | Pronounced streak-like and mixed increase in echogenicity with widespread evidence of active fascicul-ations in muscle ultrasound Denervation signs in muscle biopsy, normal EEG | Fibre type grouping of small and hyper-trophic fibres in muscle biopsy | Reduced fiber size, increased fiber-size variation, clustered type I fibers, atrophy in muscle biopsy | Fiber size variation and atrophy, type 1 fiber grouping, oxidative rims, type 1 fiber predom-inance in muscle biopsies | Intermittent bifrontal theta waves ± fronto-central sporadic epileptiform activity in EEG | Elevated creatine kinase | NA | - | Low serum biotin, muscle biopsy showed neurogenic pattern | Microcytic anemia, lactic aciduria, increased urinary 3-hydroxy-isovaleric and glutaric acids, Marginally increased CSF lactate | NA | 20% reduced urinary alpha-N-acetyl-glucos-aminidase activity |
| **Overlapping disorder** | PCH | PCD1D | - | Congenital myopathy | Congenital myopathy | CONSDIAS, PAMP Syndrome | NDD with neuro-muscular and skeletal abnor-malities | CMT2T, SCA43? | NDD with absent speech and movement and behavioral abnor-malities | SMVTD | - | Nestor-Guillermo progeria syndrome | CMT2V, MPS3B |
| **Zygosity and variants with motor neuronopathy phenotype** | Hom H923P, Hom H913R, Hom D704E, | Hom L14P, CH L14P and R161*, CH L80R and R162Lfs*3 | Hom P79R | Hom, R254*, Hom R278*, CH R254* and R278* | Hom E53Gfs19*, CH S311* and deletion of exon 5, CH R343* and 10q22.1 | Hom V335G, Hom G77T | Hom S134P, Hom Q25* | CH V440_K472 del and, P676L | CSV | Hom S429G, CH R94* and Y162C | Hom L232R | Het G16R | Het A479T |

| | ***GEMIN5*** *Kour et al, 2021*[1] | ***EXOSC9*** *Burns et al, 2018*[2] *Bizzari et al, 2019*[3] *Sakamoto et al, 2021*[4] *Dabaj et al, 2022*[5] | ***RBM7*** *Giunta et al, 2016*[6] | ***TRIP4*** *Knierim et al, 2016*[7] *Töpf et al, 2021*[8] | ***ASCC1*** *Knierim et al, 2016*[7] *Giuffrida et al, 2020*[9] *Lu et al, 2020*[10] *Rosano et al, 2021*[11] | ***ADPRHL2*** *Beijer et al 2021*[12] | ***NRCAM*** *Kurolap et al, 2022*[13] *Elahi et al, 2023*[14] | ***MME*** *Hong et al, 2019*[15] | ***UBE3C*** *Gopinath et al, 2007*[16] | ***SLC5A6*** *Holling et al, 2022*[17] | ***SLC25A21*** *Boczonadi et al, 2018*[18] | ***BANF1*** *Marcelot et al, 2023*[19] | ***NAGLU*** *Lopergolo et al, 2023*[20] |
|---|---|---|---|---|---|---|---|---|---|---|---|---|---|
| | Hom G683D, Hom S1000P, CH I988F and L1367P, CH I988F and S1311Lfs*7, CH H162R and L1367P | Hom G51R, CH L14P and R212* | | | micro-deletion, CH Arg156* and c.297-8 T>G | | | | | | | | |

**Abbreviations:** CH: Compound heterozygous; CMA: Congenital multiplex arthrogryposis; CMAP: Compound muscle action potential; CMT: Charcot Marie Tooth disease; CONSDIAS: Stress-induced childhood-onset neurodegeneration with variable ataxia and seizures syndrome; CSF: Cerebrospinal fluid; CSV: Complex structural variant**;** EEG: Electroencephalography; EMG: Electromyography; FVC: Forced vital capacity; Het: Heterozygous; Hom: Homozygous; ID: Intellectual disability; MPS: Mucopolysaccharidosis; MRI: Magnetic resonance imaging; NA: Not applicable; NDD: Neurodevelopmental disorder; PAMP: Episodic psychosis, ataxia, motor neuropathy with pyramidal signs; PCH: Pontocerebellar hypoplasia; SCA: Spinocerebellar ataxia; SN: Sensory neuropathy; SMVTD: Sodium-dependent multivitamin transporter deficiency

[a]: Later-onset cases are excluded

[b]: Cases with neuropathy as a part of complex disease spectrum are excluded

[c]: Cases described as congenital myopathy are exluded

## References


1. Kour S, Rajan DS, Fortuna TR*, et al*. Loss of function mutations in GEMIN5 cause a neurodevelopmental disorder. *Nat Commun*. May 7 2021;12(1):2558. doi:10.1038/s41467-021-22627-w
2. Burns DT, Donkervoort S, Muller JS*, et al*. Variants in EXOSC9 Disrupt the RNA Exosome and Result in Cerebellar Atrophy with Spinal Motor Neuronopathy. *Am J Hum Genet*. May 3 2018;102(5):858-873. doi:10.1016/j.ajhg.2018.03.011
3. Bizzari S, Hamzeh AR, Mohamed M, Al-Ali MT, Bastaki F. Expanded PCH1D phenotype linked to EXOSC9 mutation. *Eur J Med Genet*. Jan 2020;63(1):103622. doi:10.1016/j.ejmg.2019.01.012
4. Sakamoto M, Iwama K, Sekiguchi F*, et al*. Novel EXOSC9 variants cause pontocerebellar hypoplasia type 1D with spinal motor neuronopathy and cerebellar atrophy. *J Hum Genet*. Apr 2021;66(4):401-407. doi:10.1038/s10038-020-00853-2
5. Dabaj I, Hassani A, Burglen L*, et al*. Pontocerebellar Hypoplasia Type 1D: A Case Report and Comprehensive Literature Review. *J Clin Med*. Jul 26 2022;11(15)doi:10.3390/jcm11154335
6. Giunta M, Edvardson S, Xu Y*, et al*. Altered RNA metabolism due to a homozygous RBM7 mutation in a patient with spinal motor neuropathy. *Hum Mol Genet*. Jul 15 2016;25(14):2985-2996. doi:10.1093/hmg/ddw149
7. Knierim E, Hirata H, Wolf NI*, et al*. Mutations in Subunits of the Activating Signal Cointegrator 1 Complex Are Associated with Prenatal Spinal Muscular Atrophy and Congenital Bone Fractures. *Am J Hum Genet*. Mar 3 2016;98(3):473-489. doi:10.1016/j.ajhg.2016.01.006
8. Topf A, Pyle A, Griffin H*, et al*. Exome reanalysis and proteomic profiling identified TRIP4 as a novel cause of cerebellar hypoplasia and spinal muscular atrophy (PCH1). *Eur J Hum Genet*. Sep 2021;29(9):1348-1353. doi:10.1038/s41431-021-00851-8
9. Giuffrida P, Arpa G, Vanoli A, Di Sabatino A. Comment on Jun, S.Y.; et al. "Tumor Budding and Poorly Differentiated Clusters in Small Intestinal Adenocarcinoma" Cancers 2020, 12, 2199. *Cancers (Basel)*. Oct 15 2020;12(10)doi:10.3390/cancers12102982
10. Lu W, Liang M, Su J*, et al*. Novel compound heterozygous pathogenic variants in ASCC1 in a Chinese patient with spinal muscular atrophy with congenital bone fractures 2 : Evidence supporting a "Definitive" gene-disease relationship. *Mol Genet Genomic Med*. May 2020;8(5):e1212. doi:10.1002/mgg3.1212
11. Rosano KK, Wegner DJ, Shinawi M*, et al*. Biallelic ASCC1 variants including a novel intronic variant result in expanded phenotypic spectrum of spinal muscular atrophy with congenital bone fractures 2 (SMABF2). *Am J Med Genet A*. Jul 2021;185(7):2190-2197. doi:10.1002/ajmg.a.62219
12. Beijer D, Agnew T, Rack JGM*, et al*. Biallelic ADPRHL2 mutations in complex neuropathy affect ADP ribosylation and DNA damage response. *Life Sci Alliance*. Nov 2021;4(11)doi:10.26508/lsa.202101057
13. Kurolap A, Kreuder F, Gonzaga-Jauregui C*, et al*. Bi-allelic variants in neuronal cell adhesion molecule cause a neurodevelopmental disorder characterized by developmental delay, hypotonia, neuropathy/spasticity. *Am J Hum Genet*. Mar 3 2022;109(3):518-532. doi:10.1016/j.ajhg.2022.01.004
14. Elahi Z, Soveyzi M, Nafissi S*, et al*. Bi-allelic loss of function variant in the NRCAM gene is associated with motor-predominant axonal polyneuropathy; the second report. *Mol Genet Genomic Med*. Apr 2023;11(4):e2131. doi:10.1002/mgg3.2131

15. Hong D, Fang P, Yao S, *et al*. Variants in MME are associated with autosomal-recessive distal hereditary motor neuropathy. *Ann Clin Transl Neurol*. Sep 2019;6(9):1728-1738. doi:10.1002/acn3.50868

16. Gopinath S, Blair IP, Kennerson ML, Durnall JC, Nicholson GA. A novel locus for distal motor neuron degeneration maps to chromosome 7q34-q36. *Hum Genet*. Jun 2007;121(5):559-64. doi:10.1007/s00439-007-0348-9

17. Holling T, Nampoothiri S, Tarhan B, *et al*. Novel biallelic variants expand the SLC5A6-related phenotypic spectrum. *Eur J Hum Genet*. Apr 2022;30(4):439-449. doi:10.1038/s41431-021-01033-2

18. Boczonadi V, King MS, Smith AC, *et al*. Mitochondrial oxodicarboxylate carrier deficiency is associated with mitochondrial DNA depletion and spinal muscular atrophy-like disease. *Genet Med*. Oct 2018;20(10):1224-1235. doi:10.1038/gim.2017.251

19. Marcelot A, Rodriguez-Tirado F, Cuniasse P, *et al*. A De Novo Sequence Variant in Barrier-to-Autointegration Factor Is Associated with Dominant Motor Neuronopathy. *Cells*. Mar 9 2023;12(6)doi:10.3390/cells12060847

20. Lopergolo D, Salvatore S, Sorrentino V, Malandrini A, Santorelli FM, Battisti C. Early-onset motor polyneuropathy associated with a novel dominant NAGLU mutation. *Neurol Sci*. Apr 2023;44(4):1415-1418. doi:10.1007/s10072-023-06607-0
